# Data on students’ learning experiences in mathematics during the COVID-19 school closure

**DOI:** 10.1016/j.dib.2021.107537

**Published:** 2021-11-04

**Authors:** Angel Mukuka, Overson Shumba, Henry M. Mulenga

**Affiliations:** a African Centre of Excellence for Innovative Teaching and Learning Mathematics and Science, University of Rwanda-College of Education, Rwanda; b Department of Mathematics, Science, and Technology Education, Mukuba University, Kitwe, Zambia; c School of Mathematics and Natural Sciences, Copperbelt University, Kitwe, Zambia

**Keywords:** COVID-19, Mathematics, Learning experiences, Remote learning

## Abstract

Like in other education systems around the world, the COVID-19 school closure in Zambia necessitated a shift from physical classroom face-to-face interactions to remote learning. However, it was not clear whether all students’ remained engaged with the learning of mathematics during that time. The data described in this paper were collected to support the findings of a descriptive survey that aimed at finding out Zambian students’ experiences with mathematics remote learning. A semi-structured questionnaire was used to collect data from 367 secondary school students in Kitwe district. It was anticipated that the collected information could provide some valuable insights into remote learning experiences among secondary school students in times of a crisis such as the COVID-19 outbreak and beyond.

## Specifications Table

**Table:** 

Subject	Education
Specific subject area	Mathematics Education
Type of data	Table Text Numeric
How data were acquired	Data were collected from 367 secondary school students via a semi-structured questionnaire.
Data format	Raw
Parameters for data collection	The aim of the research whose data are described here was to find out secondary school students' remote learning experiences in mathematics during the COVID-19 school closure from 20 March 2020 to 14 September 2020 . Respondents were 174 grade 10 and 193 grade 11 students, from six selected secondary schools within the Kitwe district of Zambia. Of the 367 respondents, 178 (48.5%) were male while 189 (51.5%) were female. The ages of the respondents ranged from 13 to 21 years old (*M* = 16.92, *SD* = 1.47). The dataset contains three files namely, semi-structured questionnaire, closed-ended questionnaire responses, and open-ended questionnaire responses.
Description of data collection	After the refinement of the questionnaire, the data collection permit was obtained from the District Education Office. This was followed by data collection from September 28, 2020 to October 23, 2020. This was soon after the re-opening of schools effective September 14, 2020. Both closed-ended and open-ended questions were included in the questionnaire which was self-administered.
Data source location	City/Town/Region: Kitwe, Copperbelt Province Country: Zambia
Data accessibility	The data described here are openly available in the supplementary files attached to this paper
Related research article	A. Mukuka, O. Shumba, H.M. Mulenga, Students’ Experiences with Remote Learning during the COVID-19 School Closure: Implications for Mathematics Education, *Heliyon*[1]*.*10.1016/j.heliyon.2021.e07523

## Value of the Data

• These data provide insights into the prevailing situation regarding Zambian students’ experiences with remote learning during the COVID-19 school closure.
• The data are useful not only for Zambia's education system but also for other similar contexts worldwide. The data provide some insights into potentially effective mathematics teaching practices in times of a crisis such as the COVID-19 outbreak and other future emergencies.
• The data also provide a basis for further investigations regarding the evaluation and assessment of remote learning as one of the measures aimed at mitigating the impact of the COVID-19 outbreak on mathematics education.
• The data can serve as a basis for policy formulation regarding the provision of Information and Communications Technology (ICT) to support the teaching and learning of mathematics in Zambia and beyond.

## 1 Data Description

Following the declaration of COVID-19 as a pandemic on March 11, 2020, by the World Health Organisation, more than 186 countries worldwide decided to close learning institutions as one of the measures to prevent person-to-person transmission of the corona virus [2,3]. Zambia was not an exception as all the institutions of learning in the country closed on March 20, 2020, after the first two cases were recorded on March 18, 2020. Like many other education systems around the world, the Ministry of General Education (MOGE) in Zambia recommended remote learning for the continued provision of education to all learners. This study was conducted to understand students’ remote learning experiences during the COVID-19 school closure in Zambia.

The data described here were obtained via a semi-structured questionnaire. The questionnaire items were formulated in line with the existing COVID-19 related literature [4,5], the Zambian government's response to COVID-19 school closure, and the researcher-anticipated challenges associated with remote learning in the context of Zambia's secondary education system. The questionnaire comprised three major sections namely, demographic information, accessible mathematics learning options, and the challenges associated with remote learning. Further details about the collected data and response types have been highlighted in Table 1.

**Table 1:** Questionnaire structure.

Section	Question number	Collected information	Response type
Part I: Demographic Information	1	Respondent's gender	Closed-ended
	2	Respondent's grade level	Closed-ended
	3	Respondent's age (in years)	Closed-ended

	4(a) to 4(f)	Respondent's choices regarding the accessible mathematics learning options during the COVID-19 school closure	Closed-ended

Part II: Mathematical Learning Experiences During COVID-19 School Closure		Respondents were also requested to list other accessible learning options that were not listed in question 4	Open-ended
	5(a)	Respondent's most preferred mathematics learning option among the ones listed in (4)	Closed-ended
	5(b)	Respondent's choice of whether to continue learning using the stated option in 5(a) or not	Closed-ended
	5(c)	Respondent's reason for the response given in 5(b)	Open-ended

Part III: Challenges associated with remote learning during the COVID-19 school closure	6(a) to 6(j)	Respondent's choices of the challenges associated with remote learning during the COVID-19 school closure	Closed-ended
		Respondent's overall feelings about the COVID-19 school closure and the teaching/learning of mathematics	Open-ended

After defining all the variables regarding closed-ended questionnaire responses, the data were entered into a Microsoft Excel Spreadsheet. These data have been stored in the supplimentary files attached to this paper with the file name “Dataset_Closedended questionnaire responses”. Similarly, all the open-ended questionnaire responses for questions 4, 5(c), and 6 were extracted from each of the 367 completed questionnaires and have been stored under the file name, “Open-ended questionnaire responses”. The actual questionnaire items have also been stored under the file name “Questionnaire-Remote Learning Experiences”.

## 2 Experimental Design, Materials and Methods

A descriptive survey research design was employed to collect the data on students’ remote learning experiences in mathematics during the COVID-19 school closure [6]. Both the quantitative and qualitative data were collected as this was deemed necessary for providing an in-depth exploration of students’ learning experiences during the COVID-19 school closure.

Prior to the main data collection, a draft questionnaire was sent for validation to 18 experts via email. However, only 15 of these experts provided feedback giving a return rate of 83.3%. The experts who provided feedback comprised 2 Ph.D. students in mathematics education, 2 master's students in mathematics education, 6 college/university lecturers in mathematics and science education, and 5 secondary school teachers of mathematics. These experts were selected because of their experience with mathematics education research, and/or their experience with the Zambian secondary school mathematics curriculum. Like the instrument validation procedures employed by [7], validators of this questionnaire were requested to comment on the quality of the included items in terms of sufficiency, relevance, clarity, and coherence. After getting feedback from these validators, their suggestions and comments were analyzed and the final questionnaire was developed.

After the questionnaire refinement, a request for a data collection permit was sought from the District Education Office on September 24, 2020, alongside the questionnaire for approval. The permit was granted on September 28, 2020 and data collection commenced thereafter. A cluster random sampling method was used to select 367 students from selected secondary schools within the district.

**Fig. 1:**
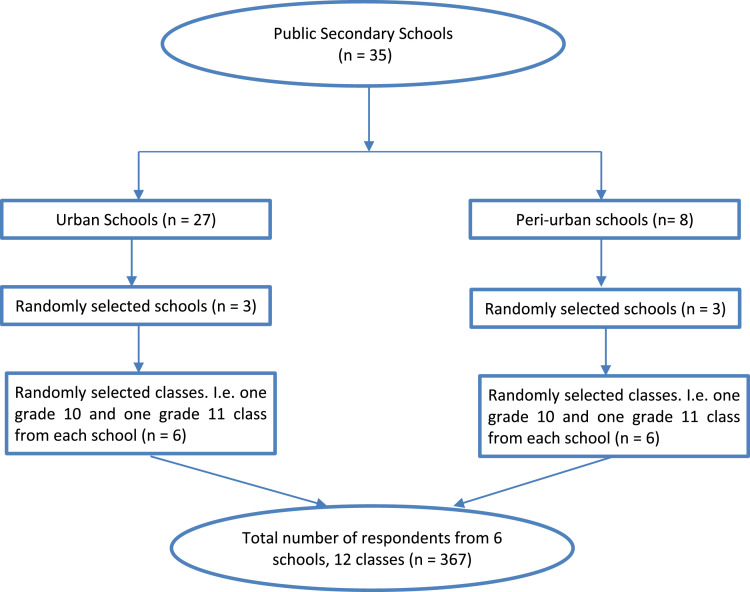
Sampling procedure.

Based on the information obtained from the district education office, schools were clustered into two categories based on their geographical locations. Schools that were located within a 10-kilometer radius from the city center^1^ were categorized as urban. On the other hand, schools located outside the 10-kilometer radius of the city center were categorized as peri-urban. Fig. 1 gives a pictorial description of how the sample was obtained while Table 2 illustrates further characteristics of the sample. The ages of the sampled respondents ranged from 13 to 21 years old (*M* = 16.92, *SD* = 1.47).

**Table 2:** Sample characteristics.

Variable	Category	Frequency	Percent
School Type	Urban	176	48
	Peri-urban	191	52
	Total	367	100

Gender	Male	178	48.5
	Female	189	51.5
	Total	367	100

Grade Level	Grade 10	174	47.4
	Grade 11	193	52.6
	Total	367	100

*Note.* School type refers to the geographical location of the sampled school based on the above-prescribed classification.

## Ethics Statement

The purpose of the study was explained to all the participants for informed consent from all of them before administering the questionnaire. Although ethics approval for this survey was not required, permission from the district education office was sought and granted.

## CRediT authorship contribution statement

**Angel Mukuka:** Conceptualization, Methodology, Data curation, Writing – original draft. **Overson Shumba:** Supervision, Validation, Writing – review & editing. **Henry M. Mulenga:** Supervision, Validation, Writing – review & editing.

## Declaration of Competing Interest

The authors declare that they have no known competing financial interests or personal relationships which have or could be perceived to have influenced the work reported in this article.

## Appendix Supplementary materials

Image, application 1

## Footnotes

1 City centre, in this case refers to the commercial and business centre of Kitwe district.
