# Supplementary material for: Data on students’ learning experiences in mathematics during the COVID-19 school closure
Source: Data Brief. 2021 Nov 4;39:107537. doi: 10.1016/j.dib.2021.107537 (PMC8601959; doi:10.1016/j.dib.2021.107537)
Supplement: Supplementary file 1 [file mmc1.zip › Open-ended questionnaire responses.docx]

**OPEN-ENDED QUESTIONNAIRE RESPONSES**

| **S/N** | **QUESTION 4** | **QUESTION 5 (C)** | **QUESTION 6** |
| --- | --- | --- | --- |
| 01 |  | **A;** Because I want to know how to answer questions in mathematics |  |
| 02 |  | **A;** Because we were not coming to school that’s why I concentrated on using books |  |
| 03 |  | **A;** Because it is much easy and less expensive | Covid-19 seriously affected me and my family because my parents struggled to find money for up-keep at home |
| 04 | Nobody was available to help me with my studies so somehow it was difficult to understand some topics by myself | Although my preferred learning mode was A, I would not like to continue learning mathematics using this mode because I need the explanation from a professional teacher for me to understand the topics clearly | It affected our learning because some of us who are slow learners needed someone to help us to understand |
| 05 |  | I would love to continue learning using C because it was easy for me to understand the topics that were not clearly taught in school. It also helped me to learn some topics that I never knew | I felt bad because COVID-19 affected my learning of mathematics and other subjects. It was not easy for me to learn the topics on my own without someone to explain. |
| 06 | I also studied with my friends at home through group work | F; because it was much easier for me to learn faster | Corona-virus affected my learning in mathematics but not that much because I was there to help myself |
| 07 |  | Although my preferred learning mode was D, I just want to continue learning the way we learn in school | I actually feel bad because I am behind and I could not even manage to answer those questions I used to solve because I forgot everything |
| 08 |  |  | I felt bad because no one was there to explain mathematical concepts to me. It affected me a lot because I was studying alone and I was not able to understand some mathematical concepts on my own |
| 09 |  | B; because it helped me a lot not only in mathematics but in other subjects also | It was very bad for me because I had to struggle to study during the Covid-19 school closure |
| 10 | No way because we were in lock down |  |  |
| 11 |  | E; because you have direct contact with the teacher and I was able to ask questions where necessary | I was affected because it wasted my time and now I have less time to prepare for my exams |
| 12 |  |  |  |
| 13 | Extra lessons | A; I would like to study alone and then get help from the teacher at school | It affected me a lot because we are now having little time to recover the lost time |
| 14 |  |  |  |
| 15 |  | E; it gives me fast understanding of things | The Covid-19 school closure had two sides: the good side is that the closure protected us from getting infected while the bad side was that it affected our learning |
| 16 | Group discussions with my friends | Although I preferred A, I would not like to continue learning in this mode because I did not know if my answers were correct or wrong because there was no one to control me whether I was right or wrong | I myself was not happy about the closure of schools because Covid-19 affected my learning of mathematics |
| 17 | Through asking my friends and relatives | A; No because it was difficult for me to understand some questions and answer them because there was no one to explain and teach me the mathematical concepts | I feel bad because it has disturbed me and has affected my mathematics lessons in school. If my parents fail to pay for my tuitions, then there will be no one to explain concepts especially that I lack mathematics text books and other learning materials |
| 18 | Group discussions with my classmates | E; because the closure took long | It has disturbed us so much because we all have little time to learn and our exams are coming soon and the attention is all going to the examination classes for now. I feel bad because the corona virus has brought me down in terms of my academic |
| 19 |  | F; yes because it was available at any time | Corona virus affected me in mathematics because the learning procedure was very tough |
| 20 |  | A; no because it is very hard to understand some concepts when you are studying mathematics on your own. Mathematics is not like other subjects where you can understand concepts without any explanation by a teacher | I felt bad because I have remained behind because of Covid-19 closure |
| 21 |  | A; no because I do not have enough material to use for learning | For me it was not good that we close school. It affected all the school going children including myself and that was painful for me |
| 22 |  | None was beneficial: Mathematics has been challenging o me but I can’t say much because it won’t help us in any way | It has been very unpleasant surprise in a bad way. It has made us to relax and fail to revise. But the good part is that some of us have learnt other things to do without finishing or completing school |
| 23 |  | A; because it was so helpful to me and I started getting used to study on my own | I felt bad because it affected my studies |
| 24 |  | E, No because private lessons usually cover half of its own syllabus while at school more explanation is given | A lot of work has not been done thereby increasing pressure. Slow learners will have difficulties in understanding the concepts of mathematics |
| 25 | None apart from studying on my own | A and E; because it helps in studying and in researching on the internet | It was bad because we are behind. So the Minister of Education should look at it especially that not everyone has access internet because of lack of talk time. |
| 26 | None apart from the above | E; because to be taught physically with a teacher is good. I can ask, participate and also a teacher can know areas where I need help | I feel terrible because I was not expecting this which affected my studies and my future |
| 27 | Yes, I used to visit my friends and solved some questions from past papers although there were some questions that were difficult we tried to answer | Not decided; because I don’t know if I was answering correctly or not. | I feel a bit bad because learning wasn’t that much. I tried to concentrate and it didn’t work out. But I also feel good that school are now open for us to learn well |
| 28 | I used to go a tuition centre to learn mathematics during the Covid-19 lockdown | I would want to continue learning using A because not everything I studied on my own was understood. I still needed help from someone with experience but it was impossible because of the Covid-19 lockdown | This Covid-19 contributed to poor understanding of mathematics especially those who are slow learners. Pupils almost forgot all the formulas in mathematics |
| 29 |  | I would not like to continue with A because I need supervision from a teacher who can explain things that I cannot understand on my own | It was a disturbing experience but somehow enabled me to catch up on areas on our syllabus where I did not understand in class.  But it has done some damage to the learning of mathematics since mathematics is a practical subject which can be done well with a teacher involved. |
| 30 |  | E; because I can’t understand or solve everything by myself. So I need someone whom I can be able to ask some questions which are difficult for me | Definitely I feel bad but there is nothing I/we can do. We just have to overcome it and the learning of mathematics during the closure was not good enough. |
| 31 |  | A; because it really helped us not to forget about maths lessons and make our minds busy through solving maths question papers and test questions | The Covid-19 pandemic really affected me and the learning of mathematics because there was no one to help me to do the maths concept |
| 32 |  | E; it will be easier to understand because I will be able to ask for further explanations where I fail to understand. Although it is possible for me to learn mathematics on my own, I still need a teacher to explain difficult concepts for me. | This really affected me because for all those six months we were not learning mathematics. I am happy that we are back again at school because society is not good for a pupil. |
| 33 | I used to consult my parents and friends on some difficult topics in mathematics. | I wouldn’t like to continue with E because I was not satisfied with what they were teaching especially that they were too busy with examination classes | It affected us in a way especially that some of our parents did not get enough income to feed families. |
| 34 |  | A; because it was a very helpful way to revise and understand what I was taught by my teacher and it was an easy way to study on my own and not home | It was very bad because it affected my studies in mathematics and other subjects. Some topics in mathematics are very difficult to understand and the closure of school made me a bit behind |
| 35 |  | I would love to continue with both A and E because I can ask questions about difficult concepts in mathematics | Not good because of the loadshadding when you want to study and you can’t use a candle to study |
| 36 |  | E; because it really helped me to understand mathematics better and it was more like a revision method of the previous lessons we did in school before the closure of schools | It really affected me not only in mathematics but with all the school activities. For instance, extra lessons that used to be conducted at school got cancelled and that made me to be behind not only in mathematics but also in other subjects. |
| 37 |  | E; this kind of learning method helped me a lot because I was learning mathematics every day and there was enough time to ask and solve questions | Closing schools in this Covid period was not good in terms of learning mathematics because maths is a difficult subject. If you do not have any one to explain, you cannot understand because lessons provided on TV were also not as effective as those provided in the classroom where you can physically interact with the teacher. |
| 38 |  | A; because it is the cheapest and easiest way to learn mathematics | Knowing that mathematics is the most difficult subject the Covid-19 school closure has really caused a lot of damage. Therefore, it really affected me especially that I had no one to explain certain mathematics components nor money to pay for extra lessons |
| 39 |  | A; Since my parents cannot afford to buy televisions or pay for extra lessons, studying on my own was a better and easier way to learn mathematics | It affected me a lot because I was far behind |
| 40 |  | A; not decided because online learning and TV lessons do not allow you to ask questions where you are not clear. But in a classroom you can ask a teacher or your friend | It was very bad and it really affected me because we lost a lot of time by closing for six months. |
| 41 |  | B; no, it is not good to study mathematics a lone because you can’t control yourself when you are wrong or right especially that no one is there to correct you when are wrong. | I felt bad because I did not have time to spend with my friends whom can come and help me to solve mathematics at home or at school |
| 42 | Studying and discussing with friends | E; no because not all knowledge and complete mathematics is taught during extra lessons | This has really affected me because it is difficult to progress in mathematics because maths is a subject that requires attention. When schools closed I missed out a lot since I had no one to help me understand topics like probability, trigonometry, and even earth geometry. |
| 43 |  | A; no, because alone I can’t teach myself properly as there were some of the topics where I needed help | The Covid-19 has made me behind with my studies |
| 44 | Through discussions with my friends | A; studying and learning on my own is very much beneficial to me because it helps me in some of my mistakes in mathematics and would like to learn more by my own because the more you do things on your own the more you know. | Although Covid-19 affected me badly, I used to try to study mathematics on my own |
| 45 |  | B; It is good because I got to learn mathematics at the comfort of my home and I used to learn at a faster rate. | Codid-19 school closure affected my learning of mathematics especially that a lot of topics have not been covered |
| 46 | I used to go for tuitions and I have continued | E; I am not really good in maths and the extra lessons I get help me a lot to add on to what I have learnt. So I would like to continue also because since I started grade 10 the mathematics teacher they gave me wasn’t that good but now they have given us really good teacher. Yes I would like to continue with extra lessons | I feel bad, because I’m really dull when it comes to mathematics so this period we had to stay in lockdown really disturbed me, because I didn’t start extra lessons right away as I stayed home for almost 2 months and I also do not have enough mathematics text books. So this Covid-19 has really disturbed my mathematical mind-set |
| 47 |  | A; because when I am trying by myself to solve maths it can be easy for me to right exams by myself. And also it is good to read text books, exam papers, test papers, and mathematics note books help us to improve | This Covid-19 school closure is not good because it has affected us with the learning of mathematics |
| 48 |  | A; because studying on my own using exercise books, papers, and past examination, I can understand more than from someone because I feel free on that. | The Covid-19 school closure was bad because at school we ask our friends or teachers where we fail to understand. So I feel like it was not good. Of course it was good studying on my own but at school we understand more |
| 49 | Going for extra lessons | A; no, it is more understandable to learn from a mathematics teacher | Covid-19 made us to remain behind. It is good we have now opened schools so that we can catch-up |
| 50 |  | C; no, because sometimes I was not understanding some things they taught and I was not able to ask or to tell them to repeat | It affected me badly because I was not able to ask anyone about difficult concept in mathematics |
| 51 | I used to go for extra lessons | E; yes because during extra lessons I used to ask questions and used to go through past examination papers which really helped me to know some of the things I never knew in mathematics | I felt bad at first but after, I started going for extra lessons |
| 52 | My mother used to teach me at home with the help of past papers | B, yes because they helped me in many ways e.g. when having those lessons online I covered other topics that I had not learnt | It was a great experience as we learned a lot of things |
| 53 |  | C; for me to learn more and understand very easily | This really affected me |
| 54 | By using Adobe reader, I downloaded a lot of books for studying mathematics | A; it was easy for me to find materials to read | It affected me in a way because there was no one to explain certain things to me |
| 55 |  | E; no, because being taught by a teacher who does not know your weakness is not easy as I was not able to understand what he was teaching | I feel Covid-19 affected me badly because I started understanding mathematics but then Covid-19 came and disturbed me a lot |
| 56 |  | E; because of irregular supply of electricity, I could not learn through media such as TV, online etc. Above all, it was not possible to ask for clarifications when learning through media. | It affected me badly because I had no one to help me in mathematics |
| 57 |  |  | I did not feel good because I am very much behind at school and in mathematics because there was no one to explain certain mathematical concepts |
| 58 |  | A; no, I did not understand things on my own | The Covid-19 was so bad that many things happened |
| 59 |  | A; no, because I can’t understand things on my own. Even those lessons televised on TV were too fast for me to understand | I did not feel happy because Covid-19 affected our education as most of us do not have anyone to pay for our extra lessons |
| 60 |  | E; no because I do not have any text book that I can use when I want to study | I did not feel well because I did not have any one to help or explain things to me |
| 61 |  |  | Am not happy |
| 62 |  | A; because it helped me to go ahead of the teacher and helped me to understand mathematics on my own as the result it made me get interested in mathematics | The Covid-19 at some point affected my studies in that certain topics in mathematics I couldn’t understand them on my own because there was no one to explain to me |
| 63 | I used to study with my elder sister who was re-writing her grade 12 exams | A; no, studying mathematics on my own is not good. I was separated from my friends because of social distancing and I had no one to ask things I did not understand. | I did not feel good because I used to spend my whole time at home. I really missed school. I am not good at mathematics and I did not have a chance to ask my teacher |
| 64 |  | A; not decided, because it was my only option to do and I did not have access to e-learning |  |
| 65 |  | A; no because I can never learn new things alone. I need someone who has full knowledge of mathematics | There are some people who do not know mathematics and they can never learn on their own. So I feel they were affected because they were not practicing but only enjoying the holiday. Myself I was affected because I always felt I needed someone. |
| 66 |  | A and E; because it is important to learn on my own and also private lessons to help me in understanding maths | I feel bad I did not understand very much some topics and formulas |
| 67 | Group discussions with friends | None; My choice is to be going to school because this type of learning affected me because I never improved with my lessons | This has made me to be poor in studying because it affected my learning and my feeling was that; it specifically made me bad because on my own I cannot understand certain concepts in mathematics |
| 68 |  |  |  |
| 69 |  |  |  |
| 70 |  | A; no, because I was not understanding what I was reading | It was not good for me. I don’t know for others |
| 71 |  | A; no, because there was nobody available to help me or to explain certain mathematical concepts |  |
| 72 |  | A; no, because I can’t know much I must have someone or a teacher to teach me where I did not know | It was bad because I did not have someone to teach me mathematics |
| 73 |  |  | I feel bad because we missed a lot especially in mathematics |
| 74 |  |  |  |
| 75 |  | C; yes, because I was alone in a room and there was no noise |  |
| 76 |  |  | Covid-19 is a strong thing and maybe it will cause poor results because we are behind |
| 77 |  | C; yes, because I learned a lot on TV despite not understanding certain things |  |
| 78 |  | A; no because I want to be helped by mathematics teacher and explained to me further | I felt bad because I missed out and my learning of mathematics during school closure affected me a little because I had no one to help me start a new topic |
| 79 |  | A, E, and F; no because costs a lot of money and the person used to come late | I feel bad because it was a bit hard to learn mathematics on my own and home was boring |
| 80 |  | C; yes I was learning through TV4 and I was benefiting because it was free as we were not paying anything | I feel bad because no one helped me. I was just on my own |
| 81 | I used to ask friends and teachers who stay near my home | A; yes because I can understand certain things on my own. But physical learning is more beneficial than television because you can’t ask questions on TV | At first it was so boring and no interest of studying but after I started asking and consulting from other people and other teachers so it became interesting |
| 82 |  | A; yes I would like to continue learning in this mode so that I can understand | I feel bad because I was studying on my own and there was no one to help me in mathematics |
| 83 |  | A; not decided because others cannot manage to understand mathematics | Covid-19 is not good for those who lack electricity at home even self-study requires light at times |
| 84 |  |  | It really affected me because I had no time to study |
| 85 |  | C; no because if you do not listen well then you cannot understand | I am not happy about Covid-19 school closure because we are now behind in many topics of mathematics |
| 86 | My uncle used to teach me | A; no because using textbooks on my own I may not understand when no one is teaching me | The Covid-19 school closure disturbed my lessons at school. Yes, I can study on my own and learn using ZNBC TV 4 but when I am not seeing a teacher face-to-face in class, I cannot understand because I cannot ask a teacher on TV |
| 87 | Ye I had my uncle on my side | A; no because using a book of my own I may not understand anything because it is difficult to understand without any teacher or someone to help me when I make mistakes | I can say that the Covid-19 disturbed my learning because we stopped learning at school and it is difficult to learn without a teacher |
| 88 |  | C; no because some of us do not have electricity at home | I feel bad because I had to stay at home without learning anything |
| 89 |  | C; no because some of us we don’t have electricity to learn through television | It was very bad for me because I did not learn very well |
| 90 |  | E; yes because I really did understand properly because she could explain to me in a very good way and where I never understood, she could teach me once again | Okay, I feel like I could not change it but to accept it that it was real but I really tried my very big best to keep myself safe |
| 91 |  | A; because it makes me to know how to solve mathematics on my own | I felt bad when we closed school |
| 92 |  | A; yest because it makes me to know how to solve mathematics on my own understanding | I felt bad |
| 93 |  | A; yes because you can read textbooks anywhere especially some of us who do not have electricity | It affected me the most reason being that I do not have a smart phone or a computer at home. It has made me to forget what I learned in school |
| 94 |  | A; yes because it helps me to understand. For instance, if the teacher was teaching in class and you didn’t understand the teaching clearly you will be able to sit and go through and try to see if you can answer the questions on your own | It really affected us in our studies of mathematics because we are really behind |
| 95 |  | A; yes because when there is no electricity you cannot learn using TV | This pandemic has affected us a lot. You can’t go to anyone to help you in your studies and it is difficult to learn on your own |
| 96 | Some of my relatives and friends who have completed school used to help me | B; yes because you can go through over and over until you understand | I feel like we have little time to learn and understand the topic that we are learning so I would really like to be given more time to learn at school |
| 97 |  | A; ye, because we are able to understand on our own through studying using text book and other materials | It affected me because without schooling, we did not enjoy mathematics |
| 98 |  |  | I was affected due to the closing of schools as I remained behind and there was no one available to explain some mathematical concepts to me |
| 99 |  |  | It’s bad for us because we were missing mathematics and other subjects |
| 100 |  | B; yes, because when you are trying to solve some questions you know and understand more |  |
| 101 | My sister used to help me | A; no because we can’t understand without someone to explain or ask as we do with teachers in school | The Corona virus has brought less knowledge in mathematics and it is very hard for me but a student I try by all means to know more about mathematics and literally have someone to help me and guide me but we need access to the teachers to help us |
| 102 |  | C; no, because learning on TV is very hard especially that you cannot ask questions when you do not understand something | It affected me a lot because I did not have access to e-learning |
| 103 | I was also taught by my aunt. She is really helping me a lot | A; no because using books of my own I may not understand when no one is teaching me how to solve maths | It is making us to go down with our learnings and we are going behind |
| 104 | My sister was helping me at home and also my friends who are in grade 12 | E; yes, because I understand more when someone is teaching reason why so that you can ask questions | I did not feel good because it was not a beneficial way of learning mathematics because I prefer someone is teaching in class |
| 105 |  | E; no, because I was not understanding and construction to what I was doing | Too bad because I was not learning mathematics |
| 106 |  | E; yes, because the more the teacher teaches me the more I get to ask questions and learn more and more | I would like to continue because I felt beneficial and learnt fast and understanding |
| 107 |  | C, no because learning on TV we cannot understand everything and we cannot even ask questions because we are learning on TV | During Covid-19 school closure I was not doing good in mathematics because of lack of someone to explain |
| 108 | My brother and my friends used to help me | E; no, lack of concentration maybe I am learning and someone starts calling me | I feel very bad because of Covid-19 because I lost a lot of time to learn mathematics |
| 109 |  |  | Covid-19 school closure was very bad because the learning of mathematics was not better to students because most of us like to help each other in school days |
| 110 |  | E; not decided because I don’t get much time for extra lessons after school as I am always tired after school |  |
| 111 |  | C; no, because of lack of electricity at home. So it was hard for me to learn | I felt bad and I missed school a lot and I give thanks for minister of health |
| 112 | My sister and mother were helping me | A; yes, because any time I want to go back and check. It also helps me to know the importance of mathematics | The Covid-19 has made a big impact on mathematics because it made most of the learners to forget what they have learned |
| 113 |  | C; yes, I used to learn a lot especially when they repeat the same lesson |  |
| 114 |  | C; no, Although it was good to learn on TV during Covid-19 lockdown, I would like to learn at school, where I am able to ask for clarification when I fail to understand something. | It was good studying at home and not moving around spreading the virus of Covid-19. It was good staying at home studying mathematics at home through television |
| 115 |  | A; not decided, because I don’t really like it | It affected me a lot |
| 116 |  | B; no, lack of electricity, not always having internet bundles, usually lack of electricity and internet access |  |
| 117 | My parents and my friends used to help me | C; no because learning in this mode we cannot have more knowledge. Before the closure of Covid-19 we usually learnt well but the outbreak of Covid-19 | I did not enjoy my learning of mathematics during the Covid-19 closure |
| 118 |  | A; not decided, because I was not comfortable with this kind of learning | It was not good |
| 119 |  | A; yes it helps me to understand and challenge myself and I learn more | It was bad because things were hard for me and when you ask someone to help you they were fearing Covid-19 |
| 120 |  | B; no, because I did not understand a lot through TV or internet. On TV I could not rewind when I fail to understand | I think it was bad that we had to close schools because of the pandemic |
| 121 |  |  |  |
| 122 |  | D; yes, because the more the teacher teaches me the more I get to understand and get to ask more questions |  |
| 123 |  | A; not decided, because am a person who can make mistakes and I need someone to help me with mathematics | Covid-19 brought a lot of confusion in mathematics. I have forgotten about some mathematics and because of loadshadding we were seriously affected |
| 124 |  | C and F; not decided, I understand math faster on TV than in class | I am already forgetting a little bit of math but I will catch up when the teacher gets serious |
| 125 |  | F; no, because I did not understand what I was learning | It has disturbed in many ways because as for myself I can’t understand on my own |
| 126 |  | A; yes, it is easy to access textbooks and examination papers from the maths department |  |
| 127 |  | A and E; not decided, at time I came across the questions which I cannot answer and the private teacher at home came only for one hour during week days. But at home I used to understand more than when I am at school | The Covid-19 has seriously affected my mathematics learning and has put a lot of stress on me |
| 128 |  | A; no, some textbooks also have mistakes358  in terms of solutions. So as a pupil who does not have a lot knowledge of mathematics will think everything is correct but in the actual sense it is not. Moreover mathematics requires essential and effective teachings so as to know | It affected my learning in mathematics as it appears to be difficult without understanding certain topics in mathematics |
| 129 |  | A and E; yes, at home I used to learn more than learning at school | Covid-19 has been a lesson I have learnt in my life and has helped me to understand myself |
| 130 | Through group discussions with some classmates | E; yes, because when learning private lessons provided by mathematics teachers is better | The Covid-19 affected us a lot because we may not be able to finish the syllabus |
| 131 |  | A and C; yes, because sometimes you can miss out the information from the teacher |  |
| 132 |  |  | Our performance in mathematics has become poor because of Covid-19 |
| 133 |  | A; yes, so that we can catch up in all the subjects where we are behind |  |
| 134 | We formed a group of 8 members in which each one of us was required to teach one topic to the group. This helped us to understand a lot of topics in mathematics | B; yes, it provided easy access to new topics and they were well explained | Although Covid-19 disturbed the normal learning it helped me to learn how to use other learning plartforms that are out there. It also increased my research ability about different topics in mathematics. |
| 135 |  | C; yes, because most of the concepts in mathematics are explained by those who are teachers, so studying becomes easier because I will know what I am supposed to know about a topic | My learning of mathematics had been very difficult during the Covid-19 school closure because I had no one to ask when it comes to some concepts in mathematics even if I tried to study on my own |
| 136 | Through group discussions with friends | A; no, studying on my own using various mathematics textbooks and other examination papers cannot really help me because there are other things I need to consult from teachers and few individuals who are highly experienced | The Covid-19 has really affected most pupils due to lack of textbooks as well as the knowledge they need to acquire from teachers. This has led to a decline of performance in schools because a lot of pupils especially the non-examination classes due to long stay at home |
| 137 |  |  |  |
| 138 | Through group discussions | F; no because Covid-19 has not yet ended | I felt bad but I just need to understand the situation because they just needed to protect us from the corona virus. And for the mathematics, we made a Whatsapp group |
| 139 |  | E; yes, because it can help me to go further in mathematics | I feel like having the private lessons in mathematics |
| 140 |  | A and C; no, because sometimes will be missing some important information that the teacher can give us that’s why we cannot continue learning like this | Our performance has become poor |
| 141 | I used to attend private tuitions at a private school | A and C; no, many people in rural areas do not have electricity in their homes so it was difficult for them to learn mathematics |  |
| 142 |  | A; no, because it was too hard for me to understand the choice I made |  |
| 143 |  |  |  |
| 144 |  |  |  |
| 145 |  |  |  |
| 146 |  |  |  |
| 147 |  |  | Not better of learning we need teacher to explain for us so that we can understand |
| 148 |  | A, B, and E; no, because sometimes we were lacking materials to use, also internet and sometimes disturbances at home | It has been quite fine though just because I want to pass my final examination |
| 149 | I used to visit friends so that we can answer some questions from past papers | A; no because there is no guidance and you cannot ask the teacher | It is very bad because when the teacher is teaching you will be able to ask where you are not clear |
| 150 |  | A, B, and C; yes, because this type of learning helped me during Covid-19 school closure. | I felt bad at first but now I feel great because it has made me to concentrate on my studies |
| 151 | I used to go to private tuitions with my friends and not only in mathematics but in other subjects also | A; yes, because this type of studying is very amazing you will be able to be exposed to exam papers and you will be using different kinds of papers and books for mathematics |  |
| 152 |  |  |  |
| 153 |  |  |  |
| 154 |  | A and C; yes, the textbooks and past papers were available and the TV was available for revised work on ZNBC TV4 channel | It is affecting my studies seriously especially mathematics |
| 155 | My brother and his friend who are currently at The Copper Belt University used to teach me | A and E; yes, I benefit more because I do not need to pay for me to learn. And another thing is that I had time to spend with my brother, and when I learn I also help friends with mathematics. | Covid-19 had advantages and disadvantages which are that some of the pupils had time to study for those who had the materials but for some it was very difficult because we depend on school learning materials |
| 156 |  | A; no, when I was passing through textbooks I had no one to ask what I don’t understand |  |
| 157 | Used to discuss with friends through social media | A; no, because in some cases during my personal studies, I came across difficult mathematical concepts that I couldn’t understand and I had no one to consult. Even the questions I attempted I was not sure whether my answers were correct. Due to these uncertainties I would not want to continue with this type of learning | Covid-19 school closure was a major setback for me as it negatively affected my learning of mathematics. My performance was greatly reduced as I had little understanding while try to study on my own some mathematics topic and I had no one to consult. |
| 158 | My elder sister who is a mathematics teacher used to help me and my father was coaching me sometimes | C and E; yes; because it had helped me a lot during the lockdown days | I was very affected but now at least things are at least going ok |
| 159 |  | A and C; yes | I feel so bad that Covid-19 affected a lot of things |
| 160 |  | F; not decided, I did enjoy using this mode but because of using zoom I needed bundles and it’s not always that I was having money to purchase bundles | It was not fair with me, because I did not enough time to learn and improve my mathematics skills. I had no one to ask when I fail to understand certain topics like functions, matrices. If had a chance to be meting my mathematics teacher, I would have improved. |
| 161 |  | A; yes, because it is very helpful, when it comes to using past papers. It is very helpful to know how they prepare exams and the guidelines to use and even textbooks, exercise books and test papers | It was not easy to study because of loadshadding and other things. |
| 162 |  |  |  |
| 163 |  | B and C; yes, because it is helpful to become a successful person in life and that affect us because sometimes we lack electricity, lack of internet connection and so on… | No better because we need a teacher to explain |
| 164 |  | A and C; yes, because the text books and past papers were available and the TV was available |  |
| 165 |  |  |  |
| 166 |  | A and C; no, because there is no possibility to ask questions, hence we miss some information |  |
| 167 |  | C; no, because there is loadshadding from morning to evening. And also learning on television you cannot ask questions if you are behind |  |
| 168 | Through discussions with my friends | A; yes, because when I am studying using textbooks and exercise books, past papers, mathematics notes and also test papers I usually understood what I was studying | It affected me seriously as I missed out a lot in mathematics |
| 169 | By attending extra lessons by relatives | A; yes, because it gives learners full time to revise and catch up where they were behind | Not better because we need teachers to explain and emphasise on what we were studying |
| 170 | Through group work with friends where we used to share what we knew | A; no, because teaching yourself is not the best. Going through past papers, textbooks, and notebooks was really not beneficial to me. I need someone to guide me on how to answer some questions | The Covid-19 school closure has affected us a lot because when we went back to our respective homes we never had serious studies and we had no one to guide us on some concepts of mathematics. Moreover, anytime you want to watch the education channel, electricity won’t be available. |
| 171 | We formed a group of four with my friends and chose topics to teach ourselves. When we fail to understand something we used to ask former schoolmates | E; yes because it really helped me to be where I am right now. It also helped me to fill some gaps I had in mathematics | The closure of school was just okay as government wanted to protect us from those who were infected. The closure also helped me to see my capabilities in mathematics and it also helped me to do remedial exercises and meet more people who know mathematics to help me in the absence of my teacher |
| 172 | By making study groups with friend but not more than five | A; yes because it helped me to revise and understand topics that I did not understand with teachers | It helped me in studying mathematics because I used to revisit where I was not clear in class and I have improved in maths |
| 173 |  | A and C; no, because Covid-19 had not yet finished | Our performance has become poor |
| 174 |  | A and C; no, because there were some topics that were very difficult for me to understand and nobody was available to explain certain mathematics concepts |  |
| 175 |  | A; yes because we were exposed to many questions since mathematics does not change | It was just a waste of time because when we look at mathematics there are many things that we need to cover |
| 176 |  | E; not decided, because it costs a lot of money to pay the private teacher for lessons |  |
| 177 |  | C; no, because I cannot manage to learn mathematics without someone to ask for more explanations | A bad closure because it disturbed more things and many pupils are now pregnant |
| 178 |  | F; no, because I needed someone to explain so that I can understand better | I felt bad because it disturbed me from learning mathematics |
| 179 |  | A; no because I was not comfortable studying mathematics on my own | It affected us so much because we did not learn |
| 180 |  | F; no because it did not benefit me so much | It affected me a lot because it is not easy to learn mathematics on my own |
| 181 |  | E; no because I did not understand the concept | Bad, because it was difficult for me to understand the kind of learning which was affected |
| 182 |  | A; no because I was not understanding on my own | I felt bad because we are now behind |
| 183 |  | A; no, because it was not easy for me to manage studying by myself | It was a bad closure because it affected me and I was not concentrating by studying without anyone to help me |
| 184 |  | A; no because I could not understand the concepts | It was a bad closure because I missed a lot of learning mathematics and I was so behind |
| 185 |  | B; yes |  |
| 186 |  | A; no, I could not understand anything on my own | I feel bad for school closure because there is no other way to learn. |
| 187 |  | A; yes, I needed to have my friend | No, because I was protected from Covid-19 |
| 188 |  | F; yes, I was able to understand the content |  |
| 189 |  | C; yes, because I benefited and there was no one making noise | It is too bad this Covid-19 affected me for learning my favorite subject mathematics |
| 190 |  | A; no, because I could not understand on my own | Bad because there was no education |
| 191 |  | A; yes, because I understood the concept | Covid-19 closure was not good because it affected our learning |
| 192 |  | B; no, because I could not understand everything by myself. I need someone to explain to me properly | It affected me because it is too bad to stay home for long period of time |
| 193 |  | C; no, because I was not understanding anything | It affected me badly |
| 194 |  | F; no because I could not understand anything as I answer questions without anyone to correct me | It was a good closure because I did not get Covid-19 during the holiday |
| 195 |  | E; no because I couldn’t understand some things | It’s bad because it affected me. I wouldn’t learn I was just staying at home and watch TV. |
| 196 |  | B; yes because I benefited with mathematics | It was very bad to me because there was no learning anything at home was very bad |
| 197 |  |  |  |
| 198 |  | None, because I couldn’t ask questions | I feel too bad to be at home |
| 199 |  | None, because I could not understand anything | I lost knowledge in mathematics |
| 200 |  | None, because I couldn’t understand anything in mathematics | I am lagging behind in mathematics |
| 201 |  | None; because I can’t ask | I feel bad because I was lost |
| 202 |  | None; because I couldn’t understand | I feel bad because I am behind in mathematics |
| 203 |  | None; because I can’t ask | I feel bad because time was lost |
| 204 |  | None; because I can’t find someone to teach me mathematics | I feel I lost knowledge |
| 205 |  | None; because I could not understand | I lost time |
| 206 |  | None; because I can’t ask |  |
| 207 |  | E; no, I don’t want to continue because I was not understanding fully because at school they explain in details | It was a bad closure because it affected be badly |
| 208 |  | F; yes, because I understood the content | It was a bad closure because some of us cannot understand like the way we understood in class |
| 209 |  | A; no, because I could not understand anything | It affected me so much |
| 210 |  | C; yes, | Covid-19 was bad because I was not going to school and to church |
| 211 |  | A; yes, because I want to learn but… | It was a bad closure because it was very difficult to pass the exam |
| 212 |  | E; no, I don’t want to continue learning at home because I was understanding | I was very affected with this Covid-19 as I was not understanding mathematics |
| 213 |  | C; yes, because I couldn’t learn anything | It was bad closure because we remained behind |
| 214 |  | None; I needed someone to help me in handling difficult questions | It wasn’t a good closure because I was in need of some help to my friends on how to answer the mathematics questions |
| 215 |  | A; yes, I liked to learn in this manner because I was understanding myself and make all the answers correctly | It was affecting me because I was not able to understand some of my questions |
| 216 |  | A; no, because I wasn’t able to understand very well | It was very bad in the sense that I wasn’t able to work on some mathematics methods by myself |
| 217 |  | A; not decided’ because I could not understand anything | It affected me very much |
| 218 |  | A; yes because it was beneficial | I feel bad |
| 219 |  | A; I like it because it was helpful | It was bad |
| 220 |  | C; yes, because it was beneficial | I feel it was very bad |
| 221 |  | None; All were not beneficial | I feel it was not good |
| 222 |  | None; nothing was beneficial | I felt bad |
| 223 |  | A; no, because there was no one to explain difficult concepts | It affected me a lot |
| 224 |  | A; no because I did not benefit a lot | I feel it was bad |
| 225 |  | C; no because I did not have a chance to ask questions | I felt bad about Covid-19 lockdown |
| 226 |  |  |  |
| 227 |  | None; it was not brief | I feel bad |
| 228 |  | C; yes because it was beneficial | It was not bad |
| 229 |  | C; not decided because I did not benefit a lot | It was bad and it affected my learning of mathematics |
| 230 |  |  | It was very bad |
| 231 |  |  | I feel so bad |
| 232 |  |  | Very bad |
| 233 |  | C; no because there was no room to ask for clarification | I felt bad because there was no one to consult in mathematics |
| 234 |  |  | I feel it was very bad |
| 235 |  | D; no, it was not beneficial | I feel it was bad |
| 236 |  | C; no, I did not benefit a lot because I cannot rewind when I fail to understand something | It affected me because there was no one to teach me mathematics |
| 237 |  | None; nothing was beneficial to me | I feel that it was very bad |
| 238 |  | A; yes, because it helped me to revise some topics | It affected me because I did not understand some topics by myself |
| 239 |  | A; no because I did not benefit a lot | I feel very bad and my performance has gone down |
| 240 |  | None; nothing was beneficial to me | I feel it was very bad |
| 241 |  | None; it was not beneficial | I feel very bad |
| 242 |  | None; it was not beneficial | I feel it’s bad |
| 243 |  | C; not decided, it wasn’t beneficial | I feel bad because my knowledge has gone down |
| 244 |  | C; no, because it wasn’t beneficial | I felt bad because my knowledge has been lost |
| 245 |  | C; not decided because it wasn’t beneficial | I felt very bad |
| 246 |  |  | To me I feel my knowledge has been lost |
| 247 |  | F; no, it was not beneficial | It was bad, I lost knowledge |
| 248 |  |  | I felt it was very bad |
| 249 |  |  | It was bad |
| 250 |  | A; yes because it was beneficial to me | I feel very bad |
| 251 |  | None; nothing was helpful to me | I feel it is bad |
| 252 |  | A; no, because we were not serious to learn at home |  |
| 253 |  | F; yes it was much easy to learn on WhatsApp |  |
| 254 |  | E; yes, I enjoyed all that and I have improved in mathematics |  |
| 255 |  | F; yes, because I was learning fast and it was easy for me | I was feeling bad because I was missing a lot of things |
| 256 |  |  | I was reading on my own but the Covid-19 is the serious disease, so it was good to stay at home to be safe |
| 257 |  | A; no, because I did not study every time due to lack of electricity | Bad because at home many of us like playing |
| 258 |  | A; no because there was nobody to help explain certain concepts in mathematics | I feel bad because Covid-19 made me to sit at home |
| 259 |  | None because we do not pay much attention on TV even on internet I do not have a big phone |  |
| 260 |  | A; no, because I couldn’t understand very well on my own so I need someone to help me | I really feel bad because mathematics needs a lot of time to learn and Covid-19 disturbed me to finish up some topics |
| 261 |  | A; no because I couldn’t understand very well on my own, so I need someone to assist me | I really don’t feel good because mathematics needs a lot of time and Covid-19 has contributed to shortness of time where our teachers are being fast to finish the syllabus |
| 262 |  | None of the above because I don’t like maths. SO I was not doing anything in mathematics | Covid-19 wasn’t affecting me because I don’t like mathematics. It’s hard to understand for me but I was really upset about other subjects not mathematics. I don’t even understand mathematics when they just rub on the board they also rub in my mind |
| 263 | By helping my friends, I was also revising | E; yes, because the work was well-summarised and the teacher put more effort to teach me because I was paying | It affected me a lot and some other friends who did not have access to the internet and electricity and not able to go for extra lessons |
| 264 | Through group discussions with friends | C; yes, I didn’t manage to get smartphone or any other way of learning. It was only through TV although we had challenges of electricity loadshadding | This was a very bad spread worldwide and it affected us mainly because there was some topics that we were about to cover but because of Covid-19 we didn’t. So it was terrible |
| 265 |  | A; not decided, As for me, I can say it’s not much better to be learning alone because when am with friends it encourages me and it makes me to work hard | I feel deeply disappointed on behalf of us pupils especially grade 11 are on danger position |
| 267 |  | A; not decided, because I don’t have enough study materials | I couldn’t understand most of what I was studying due to the lack of mathematics materials and I had no one to explain some mathematical concepts |
| 268 |  |  |  |
| 269 |  |  |  |
| 270 |  |  | I am not feeling good |
| 271 |  |  |  |
| 272 |  |  |  |
| 273 |  |  |  |
| 274 |  |  |  |
| 275 |  | A and C; yes, because during that time we were learning on TV and studying on our own. It is better to continue the same way because we won’t be affected |  |
| 276 |  | A; yes because I was very impressed for my studies | Me I was very impressed for what I was studying in my holiday and I have a lack of explanation in mathematics but I tried by all means myself |
| 277 |  | C; yes, because during holidays to help me how to learn mathematics | I feel bad because Covid-19 wasted my time in mathematics |
| 278 |  |  | I did not feel good because of Covid-19 we were affected in mind |
| 279 |  | A; yes, because I want to pass the exams and to have a good life | I am not feeling good because Covid-19 has wasted my time to go to school and learn |
| 280 |  | A; no, because the learning is not good at home because nobody available to explain mathematics | I am not feeling good because Covid-19 wasted my time of learning |
| 281 |  | A, C; yes, because by passing through the past papers, textbooks, examination papers and mathematics notes I will know a lot of things. I will also know and cover some topics which we did not cover in class | It has not been easy no one to inspire me studying all by self at home and irregular supply of electricity |
| 282 |  | A; not decided, because in some of the way mathematics to me is very difficult and even when I am learning, I don’t understand | I felt bad when I heard about Covid-19. It affected me to go to school to learn and we also stopped going to church because of Covid-19 in the country |
| 283 |  | A; yes, because by passing through the past papers, textbooks, examination papers, and mathematics notes I will know a lot of things. I will also know and cover some topics which we did not cover in class | It has not been easy. No one to inspire me to study on my own |
| 284 |  | A; yes, because it helped me to revise and learn new topics | It has not been easy as there was no one to explain thing to me and electricity was not always there |
| 285 |  | A; no, because it was very difficult for me to understand some questions during the time of Covid-19 | I wouldn’t feel good about Covid-19 school closure because it was very difficult for me and I did not have enough mathematics notes and textbooks |
| 286 |  | F; not decided | It was bad studying all by self no one to inspire me and some of our friends are pregnant because of this Corona Virus |
| 287 |  | A and C; no, because Covid-19 affected me a lot | I felt bad because I don’t do well in mathematics |
| 288 |  | A; no, because Covid-19 affected my mind | I felt bad when I heard that Covid-19 had reached Zambia and caused schools to close. I don’t have someone to teach me maths at home |
| 289 |  | A; no, because it is difficult to understand when you study maths alone | Covid-19 school closure affected me because I did not learn anything during that time |
| 290 |  | A, C; yes, because I was able to revise and learn new topics | It has not been easy no one to inspire me to study maths by myself and supply of electricity was irregular |
| 291 |  | A; not decided, because in some ways I was not understanding mathematics very well. It was difficult for me due to lack of explanation | Covid-19 affected me as a learner of mathematics because it makes me to be behind from the level that I was up to another level which is too bad. |
| 292 |  | A; no, because sometimes there is lack of concentration at home and lack of understanding, but at school I can ask the mathematics teacher where I am not clear | I feel bad because we were not going forward or catching up with mathematics and there was lack of money during the Covid-19 and lack of transportation to move from one place to another |
| 293 |  | E; yes, because it is beneficial to me reason being I understand mathematics more when there is someone to explain and correct me where I go wrong | The Covid-19 school closure affected me seriously because it was not every time that I had someone explaining the concepts to me as a result I did not understand most of the things on my own. |
| 294 |  | A; no because my answer is not correct so that I can move forward in my learning | I felt bad when I heard that Covid-19 had entered Zambia so the school has been closed. I feel bad at home I don’t have mathematics teacher |
| 295 |  | A; no, because my home I don’t have electricity and TV so I don’t study good |  |
| 296 |  | A; no because there was no one controlling me where I was wrong | Covid-19 was not much good to me because I was not studying in a full time. On the other hand, Covid-19 was good for me because I was finding money for my child I made a mistake to a girl so am in pressure too much, am going in prison because of that child |
| 297 |  | A; yes because when passing through textbook, test papers, past examinations will make us to know the topics which I have not covered in class | It has been bad. We have been lagging behind in some topics in mathematics. It brought laziness as we stayed at home for a long time |
| 298 |  | A; not decided because learning at home is more difficult than school learning | I feel bad when I heard that Covid-19entered Zambia and schools were closed. So I feel bad because at home I don’t have a mathematics teacher to teach me |
| 299 |  | E; yes, because I was so happy when they were teaching me maths in the holiday | It was bad because I wanted to learn and I was not coming to school. I loved school so much I passed grade 9 exam so I was happy to come to grade 10 but Covid-19 came and destroyed everything |
| 300 |  |  | I felt bad because studying mathematics by myself was not easy |
| 301 |  | C; yes, because it was helping me to know many things and I was learning new topics | Actually it was bad concerning this Covid-19 |
| 302 |  | A; yes because it was the cheapest way for me | I felt bad during the closure because I do understand well when the teacher is teaching |
| 303 |  | A; yes, because it helped me to know the questions that come in exams and how I can answer them | I felt bad because I did not have much time to study mathematics. And I felt bad because this disease has killed many people |
| 304 |  | A; no, because this all these types of learning are expensive and my parents cannot afford to pay | As for me I feel very bad because this disease has made my performance in mathematics to go down |
| 305 |  | A; yes, Because I did not have a chance to learn mathematics using any other  way apart from studying on my own using text books and my class notes | I feel bad because the performance has gone down due to over-staying at home |
| 306 |  | It is better you learn in class where you can ask questions | It was bad because mathematics needs more attention so it was difficult for me to pay attention sometimes |
| 307 |  | C; no, because I was not understanding well what the teacher was teaching because the teacher was fast | I am not happy about the Corona pandemic because I am behind in mathematics |
| 308 |  | C; no, because I was not understanding some of the activities that were done | I feel bad because sometimes I was not learning mathematics because of being busy of something, e.g., cooking. When learning on TV, some teachers were too fast and I did not have a chance to ask questions |
| 309 |  | A; not decided because you cannot ask questions | Covid-19 affected us in so many ways and we remained behind in mathematics |
| 310 | My sister was helping me in mathematics | A; no because when you are solving mathematics you cannot ask anyone. Not all of us had access to e-learning | Covid-19 school closure made us to remain behind because we did not learn for a long time |
| 311 | My brother who is in grade 12 was helping me in mathematics |  | I feel bad about this Covid-19 school closure because we were not learning for six months |
| 312 |  | A; no, because I did not understand certain things on my own | It is not good for me because sometimes or maybe I could not understand some mathematics |
| 313 |  | E; no, because not all of us were able to access e-learning because we do not have computers, smart phones. Self-study never worked because someone needs to explain for you to understand mathematics | The closure of schools affected mathematics lessons and we are very behind now. Staying at home led to misbehavior by some pupils |
| 314 | My brother was teaching me maths | E; yes, because my private teacher was my brother so we did maths quite often | Covid-19 is very bad because all those mathematics we were learning at school were forgotten |
| 315 |  | A; yes, because it benefited me with my maths lessons and I learned a lot in a short period of time, so I will continue to study so that I won’t be left behind. I can even solve equations that our teacher has not yet taught us | Covid-19 is bad because by now we would have learnt a lot in these months we were not coming to school and we are also behind |
| 316 |  | E; no, because of lack of electricity and television at home | During the Covid-19 it is very difficult to learning television because you cannot ask questions |
| 317 |  |  | During Corona it was very difficult to learn mathematics |
| 318 |  | A; no, because I can’t understand everything on my own | It was bad because some topics in mathematics are difficult to understand |
| 319 |  | A; yes, because I want to learn more and to revise myself from what my teacher taught me | The closure of schools was bad. But we still learnt something because not everything should be done by a teacher. Even us we can teach ourselves certain things. |
| 320 |  | C; no, because of shortage of electricity. | I feel bad about this Covid-19 school closure because we stopped learning mathematics and it took time for us to start going to school |
| 321 |  | A; yes, because it has helped me to catch up the topics I missed during the Covid-19 school closure | It was not good because it delayed us for school |
| 322 |  | C; yes, because I understand better than teaching myself. At least when someone is teaching on TV, I get to learn more ideas on how to solve mathematics and I understand it well |  |
| 323 |  | A; no, because studying on my own at home was difficult. When it comes to going through past papers some of the questions I failed to answer them because I had no one to help me, and even though I had text books I needed somebody to explain to me | This closure somehow made me to become poor in maths because for me the only way I have of getting knowledge is through the teachers at school so this closure was bad for my maths academics |
| 324 |  | A; yes, because I want to know many things in mathematics | I was not happy for what happened in maths because of Covid-19. I was not participating freely in terms of mathematics because of Covid-19 |
| 325 |  | A; no because it was difficult to understand the mathematics questions fully without someone to help and explain so that I get an idea on how to simplify some equations |  |
| 326 |  | A; no, because it is hard to understand some mathematical concepts without a teacher | Staying home for a long period of time made me to start failing to quickly understand mathematics as I was before schools got closed |
| 327 |  | C; not decided because of loadshadding (lack of electricity at home) | Bad because I had challenges in mathematics |
| 328 |  | C; yes, because it made me to learn beyond my class lesson and I have learned a lot of things on ZNBC channel | I felt bad because it brought my learning back and it killed many people in the world. And it was boring in the compound because I don’t have a phone and when electricity is gone |
| 329 |  | E; no, because it was boring to learn alone at home with a teacher. Learning becomes hard because of no friend to ask | It was bad because at home I don’t have friends who go to school. Most of my friends were not even encouraging me to study maths |
| 330 |  | A; yes, because it was easy and very affordable to a mathematics learner like me. It helped me not to forget what I had learnt in mathematics | I feel bad because most of the pupils were not studying mathematics during the Covid-19 school closure |
| 331 |  | A; no, because we were so poor in mind without going to school and learn good things like mathematics and other subjects | I was not feeling well because II was not learning I was just staying at home and became so dull. |
| 332 |  | A; no because it was difficult to understand | Covid-19 school closure was bad because we were not learning at our homes |
| 333 |  |  |  |
| 334 |  | A; yes, because am able to understand what I was studying on my own | I felt bad because it made me to remain behind in mathematics due to lack of someone to explain some mathematics to me |
| 335 | We created some study groups with my friends | A; yes, because using mathematics textbooks and past papers helps a lot to know more about how mathematics questions come and these same books can help me to get a distinction in maths | I felt very bad that the closure affected me badly. I was so behind with mathematics on how to go with them |
| 336 |  | A; no, because I don’t have much exam papers and text books | I felt very bad when the closure of schools were announced because where I stay nobody available to help explain certain mathematics concepts |
| 337 |  | E; yes, because it was easy for me to understand | I felt bad because home was boring if I want to ask a question no one at home was answering except for my teacher and was not always around to answer me. It was really bad |
| 338 |  | E; yes, I would like to be learning in such a way when knocking off because I was understanding what I was learning | It was bad during that holiday because some of the topics that I was learning at home with my teacher were supposed to be covered in school |
| 339 |  | I enjoyed none because I can’t understand many things on my own. I need some teachers to help me and I can’t even concentrate when I am studying alone. | I was not happy concerning the lockdown because of Corona virus. I am totally lost on how to solve, understand, and write and many more. I would like you to help us a lot |
| 340 |  | E; yes, because without a teacher at home I can’t understand some things on my own but if there is a teacher to guide me, I can understand more on my own | On the Covid-19 school closure, it affected us because we would have covered more topics in the syllabus. |
| 341 |  | A; not decided because some things were a little bit complicated for me to understand without an explanation | Bad because it slowed me down |
| 342 | My elder brother used to teach me and we were passing through past papers together after which he was giving me some work to do | C; yes because I was able to understand what they were teaching more than what I was studying on my own | Covid-19 school closure affected me in mathematics because my performance in term 1 and that of term 2 were totally different. It even brought laziness in solving difficult questions |
| 343 |  | C; yes, because it was helping me to understand mathematics and other subjects. I enjoyed it also because there was no one to disturb me at home. | The Covid-19 pandemic made people lazy and not studying |
| 344 |  | A; yes, because it is the only one that is simple for me to manage | I feel bad because the period of my learning was spoiled and the time that was consumed by Covid-19 breakdown affected my mathematics studying and learning because of the same Covid-19 breakdown even this time my studying is not good |
| 345 |  | A; yes, because it helps to improve our studying, and help us also to know how to go about examination questions | It has affected our studying because some of us we reached at the level of stopping to study. Other pupils were saying they will only start studying when they hear an announcement about opening schools |
| 346 |  | E; yes, because it helps me to learn things which we haven’t learned at school. So that when I learn them at school it will be like a revision | Sadly the Covid-19 has brought about a poor performance at schools. And it also brought lack of information because the things which you are supposed to ask from teachers were not asked because of the closure |
| 347 | My brother used to help me to solve mathematics | A; no, because it is not easy to teach oneself. Some subjects and topics are hard for one to understand unless a teacher explains to you | I felt bad because the one who used to explain some mathematics to me was not very clear |
| 348 | Via the study group which we had made with friends | A; no, because me as a secondary school learner got used to the way our teacher was teaching mathematics. I am used to the talking and listening way of learning. | I feel bad because we are too behind and we have so many topics that we used to cover in a very short period of time |
| 349 | Through group study with friends | A; yes, because I was able to learn how questions come in the examination. Where I was not clear, I used to ask my friends for clarification | It really affected me because I used to do a lot of house chores as a result I didn’t have much time to study |
| 350 | I used to consult fellow pupils who were ahead of me | A; no, because some questions were hard to answer and so the explanations were not clear to understand on my own and shortage of textbooks and other learning materials | I feel really bad because we were away from school for long time and we lost a lot of time to catch up and the time we have is very little because it will be very hard to cover up all the things we have not learnt |
| 351 |  | A; yes, because we were given much information by our teachers and I had books, examination papers and test papers that helped me revise and learn more | It really disadvantaged me a lot because I did not have someone to guide me through even thought I had all the necessary materials. Loadshadding affected me because I used to learn using TV at times |
| 352 | I tried to answer some questions in mathematics with my friends | C; yes, because it has benefited me a lot instead of sitting home without doing anything. I can learn more and know some things I didn’t know | It made me become more lazy and became less active in mathematics. It also affected my learning especially that I had no one available to help explain certain mathematical concepts |
| 353 | I used to ask my friends who were using e-learning and TV | A; yes, because it is easy and cheaper for me | I felt very sad because us learners we have been left behind in terms of learning mathematics. So Covid-19 bring so many troubles in all countries |
| 354 | By asking and consulting older pupils who have knowledge about some topics | A; no, because some statements and words were so confusing to understand. And the other reason is that the materials(books) were not available | The closure of schools affected me and other pupils because the time we needed to learn was spent at home were we couldn’t research in many topics compared to the time when we had been in school |
| 355 | My sister, who is a graduate used to help me. She gave me some tips of answering some questions in mathematics and gave me guidance in answering questions that I found difficult | A; no, because using textbooks and other examination papers is not suitable. Studying without an experienced person’s guidance isn’t suitable in some cases. Maybe you are studying and you find a question difficult and you fail to answer then you will never know how to answer a question | Covid-19 makes me feel bad because in some cases whereby you want to study mathematics you will need assistance and discussion with friends but because of the lockdown you will not be able to interact with your friends |
| 356 | I used to go for private tuitions | A; yes, because it may help me to cover up some topics that we have not yet covered at school | I felt bad because it made me behind to my mathematics learning. I did not cover up my mathematics topics because of it |
| 357 |  | A; no, because it is not that we understand everything. We need our teachers to help us in some way | It affected me because when it comes to maths you need someone to help you but if you don’t have, it is impossible |
| 358 |  | E; yes, private lessons provided by mathematics teachers at home was beneficial to me because I was able to ask where I was wrong and where I did not understand | I feel bad because I was very behind during the Covid-19 pandemic and I was not able to complete grade 10 |
| 359 |  | A; no, because reading text books on my own was not so beneficial. I had no one to consult or discuss with and that’s why I do not think I would like to continue with it | I think the closure had interrupted because we are behind with the topics. The learning of mathematics was not that bad but it was not 100% learning and I prefer being in school rather than studying on my own |
| 360 | We made study groups with friends | A; not decided because I don’t have enough materials for me to study on my own and even so it can be difficult for me to understand some questions | I felt bad because it affected my learning and I wasn’t going to school because of Covid-19 |
| 361 | I used to study with my friend who is in grade 11 | A; not decided because there is no adequate power supply when trying to study at night | It affected me because moving around to other places was discouraged to avoid contracting the virus and because we closed schools |
| 362 |  | E; yes, because it helps me to know more and revise well | It really disadvantaged me , I became slow in thinking but the learning of mathematics helped me during the closure |
| 363 |  | B; yes, because it helps me to revise and learn more | It really interrupted the syllabus and my study time table |
| 364 |  | A; no, because it was difficult for me to understand without any one to explain to me. I used to take time for me to understand the concept of the book | I did not feel good when it comes to studying |
| 365 | I used to consult both grade 12 and school levers on mathematical concepts where I was not clear | E; yes, It is helpful to me because I get to understand and have more information on other topics which we have not learnt and for easy revision at home or when am in school | I feel terribly bad on that because it has affected me a lot especially at school and the 6 months closure of schools which has made us to be behind on other topics |
| 366 | I used to go for private tuitions with my friends | C; yes, because it gave me a lot of understanding and when the teacher came to teach in class it was like I was revising | I felt bad because when I was learning mathematics on TV sometimes I used to lack electricity due to loadshadding. Another challenge was that there was no room for asking questions when you don’t understand something |
| 367 |  | A; no, because you cannot know the answer just by reading textbooks or going through past papers. You need someone to guide you where you are not clear | I feel bad because it affected us a lot and it is still affecting us because of the time we are knocking off. We didn’t have enough time to learn because of the same Covid-19 |
