# Supplementary material for: Data on students’ learning experiences in mathematics during the COVID-19 school closure
Source: Data Brief. 2021 Nov 4;39:107537. doi: 10.1016/j.dib.2021.107537 (PMC8601959; doi:10.1016/j.dib.2021.107537)
Supplement: Supplementary file 1 [file mmc1.zip › Questionnaire-Remote Learning Experiences.pdf]

EXPERIENCES WITH MATHEMATICS TEACHING AND LEARNING QUESTIONNAIRE

This questionnaire is meant to explore secondary school students' experiences with mathematics teaching and learning during the COVID-19 school closure. As a student, you are asked to give your personal views about your access to the available mathematical learning modes during the COVID-19 school closure. You are also requested to state the benefits and the challenges associated with the learning modes that you used. You are assured of complete confidentiality. Your views will be analysed together with those of others. Neither will your school nor your place of residence be disclosed in any of the associated publications. The information you will provide is going to be very useful for improving the teaching and learning of mathematics in secondary schools especially during a crisis such as the COVID-19 pandemic.

INSTRUCTIONS

This questionnaire has ***two*** *parts and consists of* ***two*** *printed pages. Please tick ✓, circle, or insert the most appropriate response when answering the questions. Attach additional paper if you need more space for your explanation*

We ask you to answer each question completely

Part I: Demographic Information

1. Gender (Male or female): ____
2. Grade: ____
3. Age: ____

Part II: Mathematical Learning Experiences During Covid-19 School Closure

4. Select by ticking ***'Yes'*** for each of the learning options that were accessible to you and ***'No'*** for those that were not accessible to you.

How were you learning mathematics during the Covid-19 school closure?	Yes	No
(a) Studied on my own using mathematics textbooks, past examination/test papers, and mathematics note/exercise books		
(b) Self-study using e-Learning and Smart Revision portals that were recently launched		
(c) Televised mathematics lessons on ZNBC's TV4 channel		
(d) Mathematics lessons aired on the radio		
(e) Private lessons provided by a mathematics teacher at home		
(f) Online lessons that were provided by the mathematics teachers through WhatsApp, Facebook, Zoom, etc.		

Is there any other way you continued to learn mathematics during the Covid-19 school closure? (please explain)

____

____

____

____

____

____

____

5. (a) Among the accessible learning modes in (4) above, which one was the most beneficial to you as a learner of secondary school mathematics? (Indicate **a, b, c, d, e, f** or **specify** if it is not among the ones listed): ____

(b) Would you want to continue learning in this mode in (5a)? (Indicate **Yes**, **No**, or **Not decided**):____

(c) Briefly give reasons for your choice in (b) above:

____
____
____
____
____
____

6. Indicate whether or not each of the following challenges affected your learning of mathematics during the Covid-19 school closure using the following criteria:

1= not affected, 2 = affected

Did the following affect your learning of mathematics during the Covid-19 school closure?	***Choice***	
(a) Lack of electricity at home	*1*	*2*
(b) Irregular supply of electricity (load shedding)	*1*	*2*
(c) Lack of a television set at home	*1*	*2*
(d) Lack of radio at home	*1*	*2*
(e) Lack of ICT gadgets, like smartphones and computers at home	*1*	*2*
(f) Irregular subscription for TV channel providers such as Topstar, Gotv, Dstv, etc.	*1*	*2*
(g) Lack of mathematics textbooks and other learning materials	*1*	*2*
(h) Nobody available to help explain certain mathematical concepts	*1*	*2*
(i) Lack of access to the internet	*1*	*2*
(j) Limited access to the internet	*1*	*2*

Overall, how do you feel about the Covid-19 school closure and your learning of mathematics?

____
____
____
____
____
____
____
____

THANK YOU FOR YOUR PARTICIPATION!
